# Multifoci and multiserotypes circulation of dengue virus in Senegal between 2017 and 2018

**DOI:** 10.1186/s12879-021-06580-z

**Published:** 2021-08-24

**Authors:** Idrissa Dieng, Marie Henriette Dior Ndione, Cheikh Fall, Moussa Moïse Diagne, Mamadou Diop, Aboubacry Gaye, Mamadou Aliou Barry, Boly Diop, Mamadou Ndiaye, Abdoulaye Bousso, Gamou Fall, Cheikh Loucoubar, Oumar Faye, Amadou Alpha Sall, Ousmane Faye

**Affiliations:** 1 Arboviruses and Haemorrhagic Fever Viruses Unit, Virology Department, Institut Pasteur de Dakar, 220, Dakar, Senegal; 2 Epidemiology, Clinical Research and Data Science Unit, Institut Pasteur de Dakar, 220, Dakar, Senegal; 3 Prevention Department, Ministry of Health, Dakar, Senegal

**Keywords:** Genetic diversity, Dengue virus, Senegal, Serotype 1, Serotype 2, Serotype 3, Co-circulation

## Abstract

**Background:**

Dengue fever is a mosquito born disease associated with self-limited to life threatening illness. First detected in Senegal in the nineteenth century, and despite its growing incidence this last decade, significant knowledge gaps exist in our knowledge of genetic diversity of circulating strains. This study highlights the circulating serotypes and genotypes between January 2017 and December 2018 and their spatial and temporal distribution throughout all regions of Senegal.

**Methods:**

We used 56 dengue virus (DENV) strains for the analysis collected from 11 sampling areas: 39 from all regions of Senegal, and 17 isolates from Thiès, a particular area of the country. Two real time RT-qPCR systems were used to confirm dengue infection and corresponding serotypes. For molecular characterization, CprM gene was sequenced and submitted to phylogenetic analysis for serotypes and genotypes assignment.

**Results:**

Three dengue virus serotypes (DENV-1–3) were detected by all used methods. DENV-3 was detected in 50% (28/56) of the isolates, followed by DENV-1 and DENV-2, each representing 25% (14/56) of the isolates. DENV-3 belongs to genotype III, DENV-1 to genotype V and DENV-2 to Cosmopolitan genotype. Serotype 3 was detected in 7 sampling locations and a co-circulation of different serotypes was observed in Thiès, Fatick and Richard-toll.

**Conclusions:**

These results emphasize the need of continuous DENV surveillance in Senegal to detect DENV cases, to define circulating serotypes/genotypes and to prevent the spread and the occurrence of severe cases.

**Supplementary Information:**

The online version contains supplementary material available at 10.1186/s12879-021-06580-z.

## Background

Dengue fever (DF) is the most prevalent arboviral disease worldwide [1]. Dengue virus (DENV) is transmitted to human through the bite of infected mosquito vectors of *Aedes* genus [2]. More than the third of the world’s population is at risk of DENV infection [1]. Over the past 50 years, DENV incidence has increased 30-fold, associated with continued geographic expansion [3]. Approximately 3.6 billion people are estimated to be at risk of dengue infections worldwide [4], apparent cases overally range from 50 to 100 million per year [5]; fatality is estimated in 10,000 deaths per year [6]. The most affected areas are America, South-East Asia and regions of Western Pacific [2]. In Africa DENV is known to circulate since the nineteenth century but due to the lack of diagnostic tools and effective surveillance, the real burden is likely to be underestimated [3, 7].

DENV infection can cause a variety of clinical manifestations ranging from self-limited form referred as DF to life threatening disease known as severe dengue [2].

The causative agent of dengue fever, is an enveloped, positive, single stranded RNA virus belonging to the *Flaviviridae* family, *Flavivirus* genus [8]. Its 11 kb genome contains a single open reading frame encodes three structural proteins [capsid (C), membrane (M) and envelope (E)] and seven non-structural (NS) proteins (NS1, NS2A, NS2B, NS3, NS4A, NS4B and NS5) [8].

DENV is classified into four antigenically and genetically distinct serotypes sharing around 65% of genome similarity namely DENV-1, DENV-2, DENV-3 and DENV-4 circulating worldwide [9]. DENV-1 presents five genotypes (I, II, III, IV, and V); DENV-2 is divided in six genotypes (Asian I, Asian II, Cosmopolitan, American, American/Asian and Sylvatic); Four genotypes were identified for DENV-3 (I, II, III, and V) and DENV-4 (I, II, III, Sylvatic) [10]. Genotypes are defined as strains having up to 6% divergence at a nucleotide level [10, 11].

For DENV serotyping and genotyping, several genomic regions such as the Envelope (E), E-NS1 junction and Capsid pre-membrane (C-prM) have been widely used [12]. Genotyping using a single primer set, for both amplification and sequencing, targeting the CprM gene is preferred as it is faster and cost effective [12, 13].

However, due to the absence of effective antiviral therapy, a safe vaccine that can induce efficient and balanced immune response against all different DENV serotypes/genotypes is urgently needed [14]. Genetic diversity among serotypes and genotypes can hamper vaccine development [11]. It is crucial to perform the surveillance of circulating genotypes in a given area to guide the choice of appropriate prophylactic measures before the implementation of any vaccination trial. Also, it is well known that different DENV serotypes or genotypes may trigger various immune responses [15], with heterogeneous ability to infect different target cells and cause the severe form of dengue [16].

Despite the fact that DENV and its vector are known to be present in Subsaharan Africa, only few studies have described circulating serotypes and/or genotypes [17–20]. Several studies focusing on the genetic diversity of circulating DENV were undertaken in Colombia [21], Nigeria [19] and India [22]. In Senegal, the first dengue infection was reported in 1970 [23]. Since then, many outbreaks and sporadic cases were reported [24–26]. Between 2017 and 2018, an unprecedented number of cases associated to this viruses (DENV 1–3) occurred in Senegal [27, 28]. Despite this growing incidence, the circulating serotypes and genotypes, and their distribution across the country are still unknown.

To address this gap on knowledge, we carried out a retrospective study to identify circulating DENV serotypes and genotypes among strains collected between 2017 and 2018 across the Senegal using qRT-PCR, sequencing and phylogenetic analysis. We also determined the spatial and temporal patterns of serotypes/genotypes around the country.

## Material and methods

### Study design, settings: presentation of syndromic sentinel surveillance network in Senegal (4S Network)

**Fig. 1:**
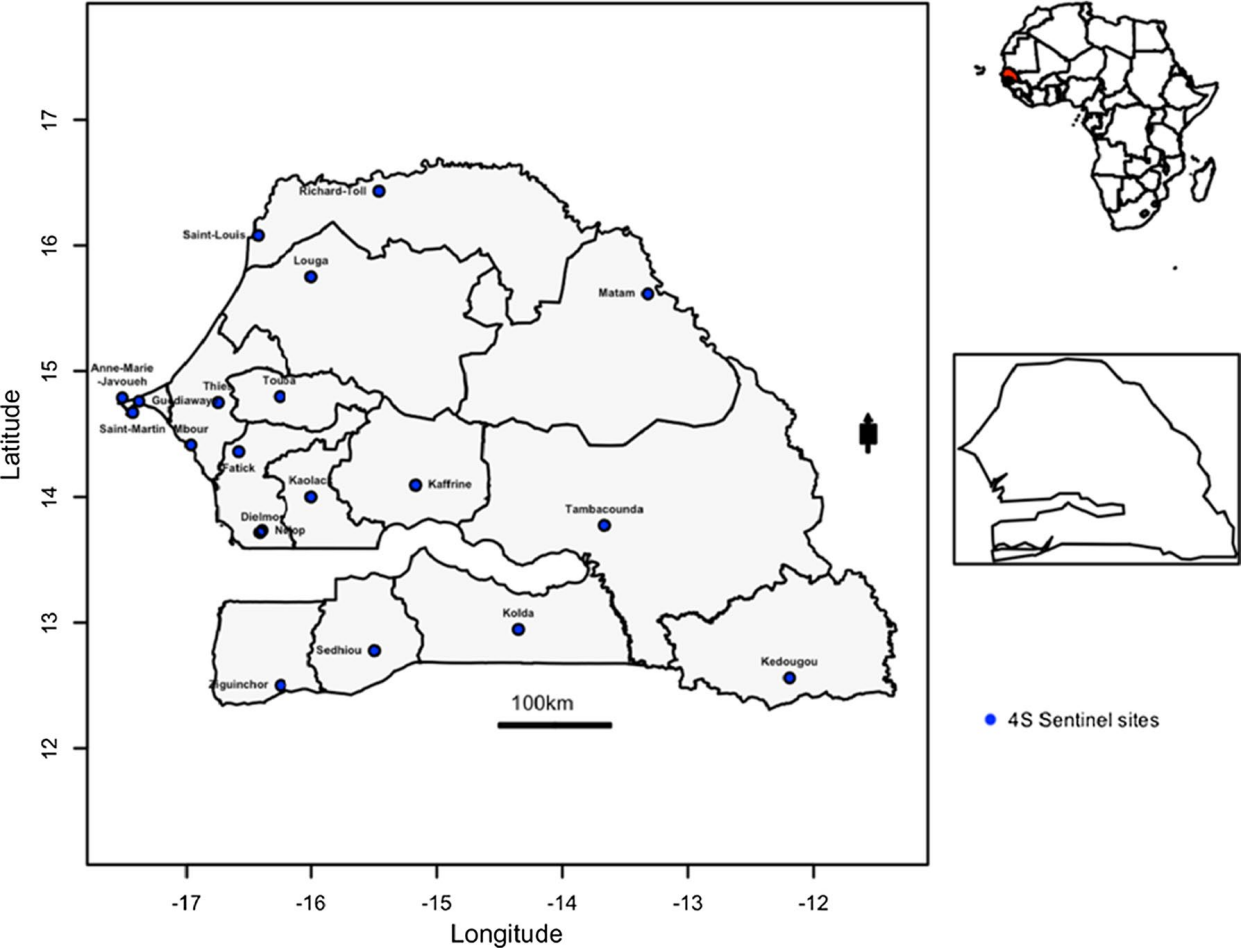
Senegal map with sentinel sites implemented for the fevers surveillance. Data from sentinel sites represented in blue dots are included in the present study

Senegal, a sub-Saharan Africa country, has long standing febrile illnesses surveillance system. Through a partnership between the Senegalese Ministry of health, the WHO country office and the Institut Pasteur de Dakar (IPD) which hosts the WHO Collaborating Center for Arboviruses WHOCC for arboviruses and the National Influenza Center [29, 30] a febrile illnesses surveillance network was established. This system, initially limited to virological surveillance of influenza like illnesses (ILI), was reviewed through the establishment of a new surveillance network, based on a syndromic approach based on fever, called Senegalese Syndromic Sentinel Surveillance Network or 4S network. The 4S network is responsible of the surveillance of febrile illnesses with 20 sentinel sites distributed in the 14 Senegal’s administrative regions [30]. These sentinel sites conduct population-based surveillance for Influenza-like illnesses and other public health priority syndromes (malaria, dengue-like syndromes and diarrheal syndromes). Outpatient visits are distributed geographically in diverse areas across the country (Fig. 1). DENV isolates were primarily derived from human samples collected between 2017 and 2018 in community healthcare centers that are part of the 4S network.

### Sera samples and data collection

From January 2017 to December 2018, in collaboration with the healthcare professionals at different sentinel sites in Senegal, patients with history of fever lasting 2 to 7 days along with one or more of the following symptoms: headache, myalgia, arthralgia, ocular pain, generalized fatigue, cough, nausea, vomiting, sore throat, rhinorrhea, difficulty breathing, diarrhea, or bleeding were offered the opportunity to enrol in the study. After informed consent was obtained, 5 ml of venous blood was collected. For each patient, standardized interview form was fulfilled containing both clinical and demographic data. The collected samples were stored at 4 °C before shipment to the WHOCC located at IPD. Samples were shipped on a weekly basis. Of note, all patients were retrospectively interviewed in the context of a sentinel febrile illnesses surveillance. At the central laboratory, collected sera were processed immediately for several arboviruses and haemorrhagic fever viruses as DENV, CHIKV, YFV, ZIKV, RVFV, CCHF by real time RT-PCR using a protocol described by Dieng and colleagues [25]. Aliquots of sera samples were also stored at − 80 °C for additional analysis (isolation and/or molecular characterization).

### Nucleic acid extraction and arboviruses detection

Viral RNA was extracted from 140 μl of human sera using a QIAamp viral RNA kit (Qiagen, Hilden, Germany) according to the manufacturer’s instructions. The extracted RNA was eluted in 60 μl of elution buffer and stored at − 20 until use.

Arboviral detection was assessed by real-time qRT-PCR assays for DENV [31], CHIKV [32], RVFV [33], YFV [34], ZIKV [35] using the Quantitect Probe RT-PCR Master Mix (Qiagen). Briefly, the detection was performed using ABI7500, using the following temperature profiles for all RT-qPCR assays: RT at 50 °C for 10 min, activation at 95 °C for 15 min and 45 cycles of 2-step PCR—at 95 °C for 15 s and 60 °C for 1 min.

### Viral isolation

In December 2018, 65 DENV-positive sera collected between January 2017 and December 2018 in the sentinel febrile disease surveillance setting and previously evaluated by real-time RT-PCR were available.

Viral isolation was attempted for the purpose of biobanking and futures studies.

Briefly, 200 µl of each sample diluted 1:10 in Leibovitz 15 (L15) medium in a 25 cm^2^ flask of C6/36 cells at 80% confluence, followed by incubation at 28 °C for 1 h to allow virus binding and entry. After incubation, L15 medium containing 5% FBS, 1% penicillin and streptomycin, 0.05% fungizone was added to the flask and incubated for 8 days according to the protocol described by Dieng and colleagues [36].

The culture supernatant was collected on day 8, to assess viral infection, Immuno Fluorescence Assay (IFA) was performed as previously described [37]. If DENV isolation failed at passage 1, the supernatant from the previous passage was used to infect a new batch of C6/36 cells as above. This was repeated up to three times until viral isolation is achieved.

Isolation was successfully achieved on 39 of the 65 sera samples used (Table 1).

**Table 1:** Summary of successfully isolated strains among DENV qRT-PCR positives sera collected during the study period

Sites of sera sampling	PCR positives (Number of successfully isolated strains)
Louga	11 (11)
Rosso	10 (07)
Matam	5 (1)
Richard-Toll	7 (3)
Bokidiawe	5 (1)
Fatick	4 (2)
Touba	10 (10)
Thiès	2 (1)
Tambacounda	5 (1)
Dakar	4 (1)
Koki	2 (1)

Once isolation was confirmed, the contents of the vial were transferred to a 15-ml tube and clarified by low-speed centrifugation (2500 rpm) at 4 °C for 5 min. The supernatant was collected and stored at − 80 °C until further use.

### Serotyping of isolates

To assess the serotypes of the successfully isolated strains, real-time molecular serotyping was performed on the RNA extracts from viral isolates using the commercial Tib-Molbiol Modular Dx Dengue Typing Kit (Cat-No. 40-0700-24) and the Lightcycler 480 (Roche, Penzberg, Germany). This system allows simultaneous detection of four dengue virus serotypes from 5 μl of input RNA [26].

### Molecular characterization of CprM gene

For cDNA synthesis, 10 μl of viral RNA (from cell culture supernatant) were mixed with 1 μl of the random hexamer primers (2 pmol) and the mixture was heated at 95 °C for 2 min. Reverse transcription was performed in 20 μl mixture containing mixed of 2.5 U RNasin (Promega, Madison, USA), 1 μl of deoxynucleotide triphosphate (dNTP) (10 mM each DNTP), 5 U of AMV reverse transcriptase (Promega, Madison, USA) and incubated at 42 °C for 60 min. PCR products were generated using set of primers DS1/DS2 described by Lanciotti and Colleagues [19] at the concentration to amplify the CprM gene. Five microliters of cDNA were mixed with 10× buffer, 3 μl of each primer at 10 µM, 5 μl of dNTPs 10 mM, 3 μl of MgCl_2_, and 0.5 μl of Taq polymerase (Promega, Madison, USA).

The obtained amplicons were purified using a QIAquick Spin PCR Purification kit (Qiagen, Hilden, Germany) normalized to 2 ng/µl then at least 10 µl of each amplicon sent for bidirectional sequencing with an ABI PRISM 377 automated sequencer (Applied Biosystems) using 5 µl of each of the same PCR primers at the concentration of 5 µM.

The raw data was then sent to the laboratory for analysis. Chromatograms were analyzed using CodonCode Aligner 3.7.1 (Codon Code, Center Ville, MA, USA) with a Phred quality score cut off of 20 as the cut-off for low-quality sequence trimming.

Obtained cleaned sequences were then merged using EMBOSS Merger software and final results were analyzed using the Basic Local Alignment Search Tool (BLAST, www.ncbi.nlm.nih.gov/) consulted on 18 July 2019. BLAST analysis was performed in order to define proximity with globally sequenced dengue strains deposed in Genbank.

The percentage of identities were obtained using nBLAST [38]. The similarity of the sequences generated during this study with those previously submitted in GenBank was determined by percentage identity and E-value, and only the best hits were used.

Cleaned CprM sequences were combined with 17 other ones from Thiès 2018 in Senegal and already available on Genbank. The obtained final dataset of 56 CprM genes was combined with DENV sequences representing the different serotypes/genotypes across the world manually curated using geneious prime 2021.0.2 (Biomatters, New Zealand), and alignment was made using Mafft [39].

Maximum likelihood (ML) trees were inferred for serotypes and genotypes determination using IQtree software [40]. All sequences were presented in the format: lab number_location_year of isolation in the phylogenetic trees.

### Spatial mapping of defined DENV serotypes

According to the results obtained from the serotyping (qRT-PCR, phylogenetic analysis), maps of Senegal were drawn using Maptools package implemented in R. For each isolate respective XY coordinates of sentinel sites regions’ where corresponding sera samples were collected were used for georeferencing.

Each described serotype at a given sentinel sites was represented by a single-coloured dot respectively as follow: yellow for DENV-1, blue for DENV-2 and green for DENV-3. At each coordinate, there will be as many points as circulating serotypes.

### Ethics statement

Samples used in this study are part of the Institut Pasteur de Dakar collection (WHO Collaborating Centre for Arboviruses and/or Haemorrhagic Fever Reference and Research). Therefore, all the samples were anonymous and only reference numbers were used during the analysis. The Senegalese National Ethical Committee of the Ministry of Health approved the surveillance protocol which lead to the obtention of human sera as less than minimal risk research, and written consent were not required. Throughout the study, the database was shared with the Epidemiology Department at the Senegalese Ministry of Health and Prevention for appropriate public health action.

## Results

### Characterization of identified dengue isolates

**Fig. 2:**
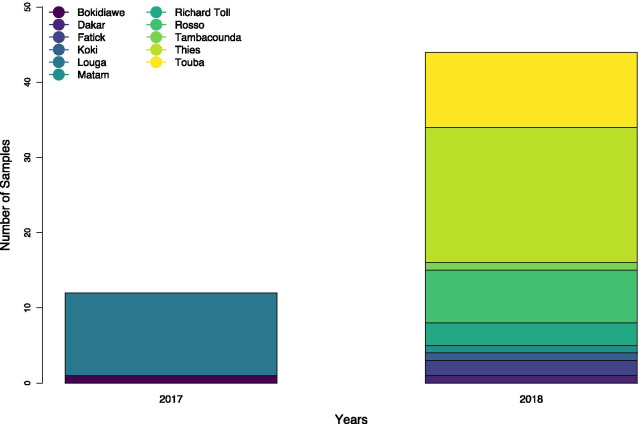
Barplot showing the distribution of used strains according to the year of detections. Used isolates were collected between 2017 and 2018, collection sites are colored according to the sampling location (cf legend)

During this retrospective study, a total of 56 (39 newly sequenced) dengue virus isolates were successfully serotyped using both sequencing of CprM gene followed by phylogenetic analysis and/or real time RT-qPCR. The overall distribution of identified DENV serotypes during this study across sampling locations in the 4S sentinel site network (Fig. 1) are represented in Fig. 2.

Demographic (sampling locations, years of isolation, age, gender) and Clinical data associated with newly sequenced isolates during this study are summarized in Additional file 1: Table S1.

### Detected serotypes/genotypes and their spatio-temporal repartition

**Fig. 3:**
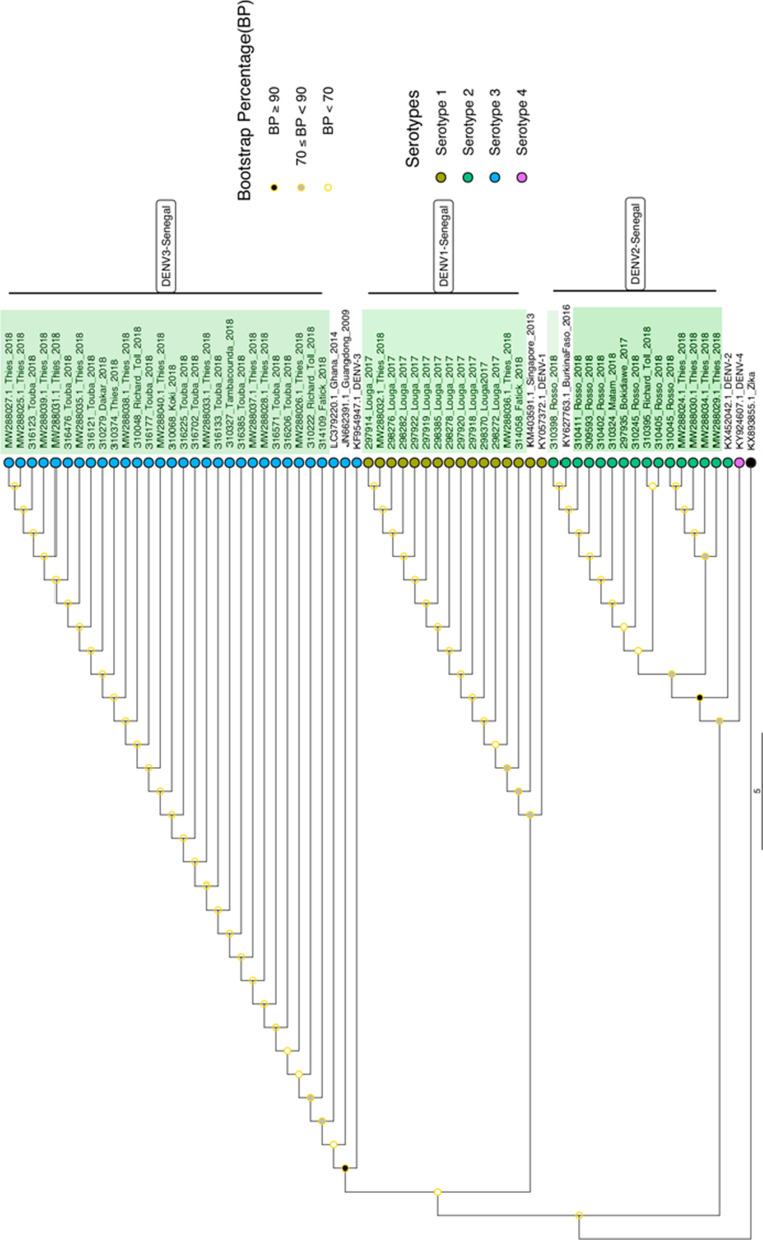
Maximum likelihood (ML) phylogenetic tree based on CprM gene drawn using Iqtree [22] assessing serotypes and genetic relatedness of Senegalese strains obtained during this study (highlighted in green) with Genebank available strains. The used evolutionary model was HKY + F + G4. Tips were colored according to the serotypes. 1000 iterations of the sequences data were used for the robustness. Bootstrap confidence cut off (Bootstrap Percentage) are shown at each node. Nodes with bootstrap values > 70 are considered to be well supported. Zika virus (KX893855) was used as outgroup

For a total of 56 serotyped isolates during this study the number of detected DENV serotypes vary by years and location (Additional file 1: Figure S1; Table S1). Overall, three dengue virus serotypes were detected (Fig. 3). No cases of DENV serotype 4 (DENV-4) were observed. At a genotypic level, phylogenetic analysis based on CprM gene reveal that dengue 1 sequences obtain during this study (n = 14) grouped into genotype V (Additional file 1: Figure S2), DENV-2 (n = 14) into genotype Cosmopolitan (Additional file 1: Figure S3) and DENV-3 (n = 28) into the genotype III (Additional file 1: Figure S4).

**Fig. 4:**
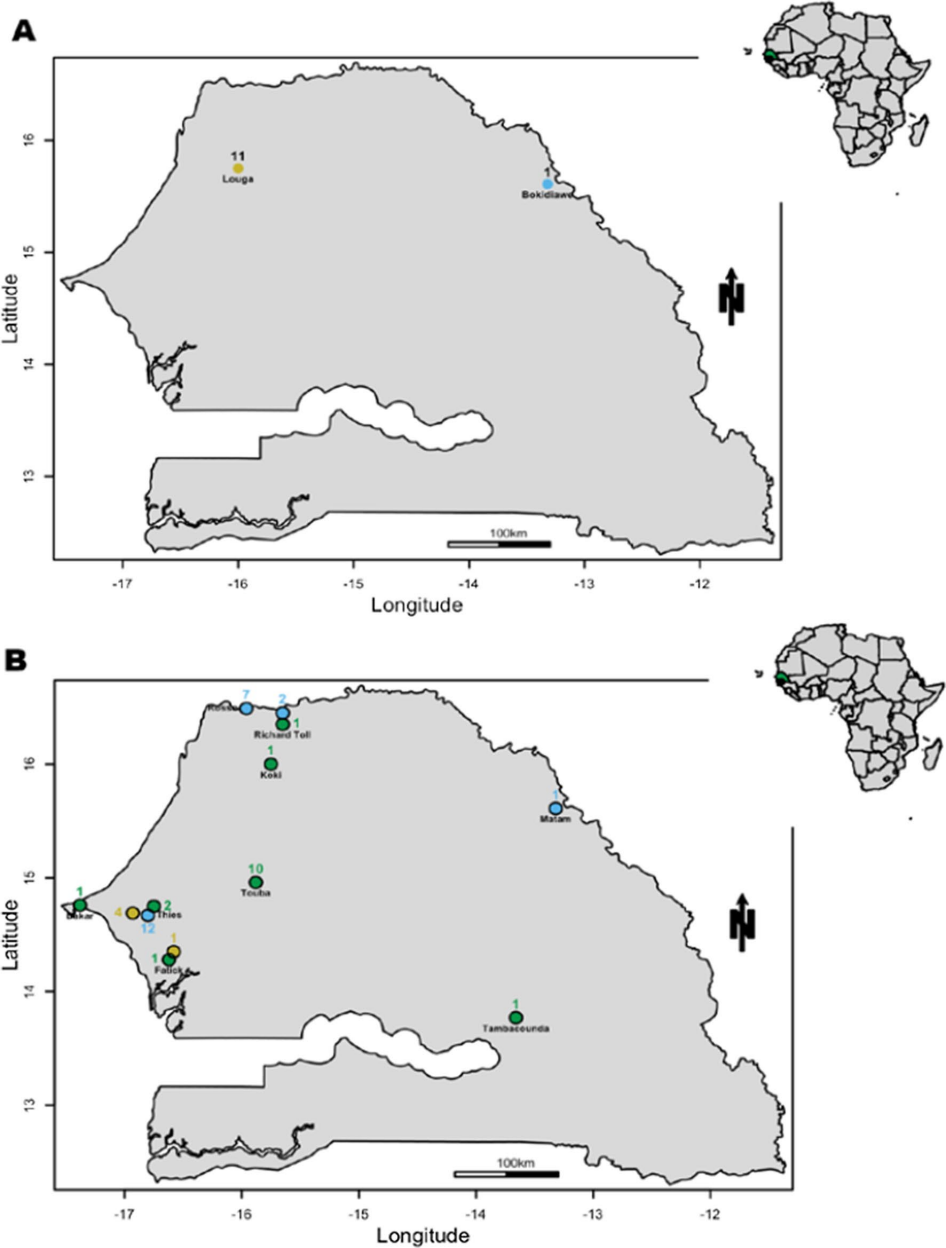
Map showing the spatio-temporal distribution of Dengue serotypes circulating in Senegal in 2017 (**A**) and 2018 (**B**). Yellow circle corresponds to dengue virus serotype 1, blue circle for dengue virus serotype 2, green circle for dengue virus serotype 3. The numbers indicate the number of serotyped isolates from a given area

Based on the informations available in Additional file 1: Table S2 and the defined serotypes (qRT-PCR and/or CprM sequencing followed by Phylogenetic analysis), we drawn maps of Senegal showing the spatio-temporal distribution of defined serotypes covering the study period (Fig. 4).

According to the obtained maps, in 2017 among 12 serotyped isolates DENV-1 was detected in Louga (11/12; 91.66%) and one DENV-2 strain in Bokidiawe area (1/12; 8,33%) located in Matam region (Fig. 4A). In contrast in 2018 among 44 serotyped isolates a co-circulation DENV1-3 were noticed in 9 localities belonging to 8 distinct administrative regions (Fig. 4B).

Considering the overall study period, DENV-2 circulated mainly in the North (Rosso, Matam, Richard Toll, Bokidiawé), DENV-1 in Louga, Fatick and Thiès, and DENV-3 was observed in seven distinct localities around the country.

Using both methods, the predominant serotype was DENV serotype 3 (DENV-3) accounting for 50% (28/56) of serotyped strains, followed by DENV serotype 1 (DENV-1) with 25% (14/56) and then dengue virus serotype 2 (DENV-2) in 25% (14/56) of studied strains.

### BLAST results

Genetic similarity of Senegalese DENV isolates compared to other global types shows that DENV-1 is closely related (99.18% nucleotides) to an isolate from Singapore (KM403584.1) in 2012, DENV-2 shares closer identity (100% nucleotides) with a strain isolated in the 2016 outbreak in Burkina Faso (KY627763.1) and finally DENV-3 has 99.64% identity with a strain isolated in China (JN662391.1) in 2009 and also in Ghana (LC379221.1) in 2014.

## Discussion

To our knowledge, this work represents the first nationwide study assessing the genetic diversity of DENV strains circulating in Senegal at the genotype and serotype level. The results of this study shows that between 2017 and 2018, three DENV serotypes (1, 2 and 3) were co-circulating in 11 localities belonging to eight administrative regions of Senegal. Co-circulation of multiple serotypes has been commonly reported in Asian countries such as Malaysia [41], India [22] and America [42]. The co-circulation of three different dengue virus serotypes in Africa was first described during the 2007–2010 dengue epidemic reported in Gabon [43]. Recently, DENV-2 and DENV-3 co-circulated during an epidemic in Burkina Faso in 2016, with DENV-2 representing the dominant serotype [44].

Co-circulation of multiple dengue virus serotypes in the same area is known to be a risk factor for the emergence of severe dengue [45]. Indeed, antibody-dependent enhancement (ADE) can occur in areas where different serotypes are circulating [22].

This highlights an urgent need for enhanced disease surveillance and the application of a vector control policy to reduce the vector density that determines the intensity of transmission.

Compared to Asian and Caribbean regions, the epidemiology of dengue in Africa is not well understood. Indeed, the lack of surveillance and reliable diagnostic tools result in underestimation of the real burden of the disease in African countries [7].

As soon as data about dengue epidemiology is available, there are sparse information regarding the circulating serotypes and genotypes.

Yamashita and colleagues report the limited numbers of studies describing the dengue virus serotypes/genotypes occurrence in Sub-Saharan Africa where the virus and the vector are widely distributed [18]. This lack of informations about circulating serotypes/genotypes is linked to the low amount of sequences from Africa in public databases as Genbank.

Among the described serotypes during this study, DENV-3 was the predominant and the most geographically widely distributed serotype across Senegal during our study period. This is particularly interesting since the latest documented occurrence of DENV-3 in Senegal was during an unprecedented urban epidemic in 2009 with 196 confirmed cases [24]. Nine years after, this serotype re-emerged in 2018. Indeed, the Senegalese ministry of health in collaboration with the Institut Pasteur de Dakar reported an DENV-3 outbreak in October 26, 2018 in Touba city [27]. Interestingly, the outbreak occurred during the Grand Magal celebration, a religious event considered to be one of the largest Muslim pilgrimages/mass gatherings in West Africa, which is an occasion for scattered families around the country (and even around the world) to gather in Touba.

Diagne and colleagues [28], Soxna and colleagues [27] raised concern about a potential spread of dengue virus to none affected areas around Senegal and Africa after the Magal. Here, based on highest sequences similarity among all sequenced DENV-3 a potential spread across the country as the probable consequence of the religious event is highly suspected. But more detailed study including larger genomic effort (more isolates and full genome sequencing) in combination with epidemiological data are needed to get insight about the origin and the dispersal pattern during this religious event. Otherwise this serotype is known to be endemic in West Africa [19, 46].

In terms of occurrence, DENV-3 is followed by DENV-1 detected in 14 out of 56 serotyped isolates and found in the regions of Louga (11/14), Fatick (1/14) and Thiès (2/14) where DENV outbreaks were reported in 2017 and 2018 respectively [47]. Phylogenetically, the Louga, Fatick and Thiès strains are identical and closely related (99.18% nucleotides) to a DENV strain from Singapore (KM403584.1). Based on the complete E-gene sequences, Dieng and colleagues reported that the Louga 2017 and Fatick 2018 DENV-1 isolates fall into a monophyletic cluster and were introduced into Senegal (Medina Gounass) from Singapore in 2014 (95% HPD = 2012.88–2014.84) [36].

Finally, DENV-2 isolates have been detected mainly in the north of the country (Rosso, Matam, Richard Toll and Bokidiawé) at the border with Mauritania. The DENV-2 sequences in our study (14/56; 25% of isolates) are closely related to those responsible of the 2016 epidemic in Burkina Faso (KY627736.1) [46]. Numerous imported cases of the epidemic strain from Burkina Faso have been reported outside the continent in countries such as France and Japan [46, 47], our study adds further evidence of the spread of the strain in West Africa as previously highlighted by Amoako and colleagues in their study focusing on the etiology of acute febrile illness in Ghana [48].

For genotype assignment, phylogenetic analysis of sequenced CprM gene during this study revealed that all DENV-1 belongs to the genotype V, DENV-2 to the Cosmopolitan genotype, while all sequenced DENV-3 during this study fall in the genotype III. Our results is concordant with the overall genotypes distribution in Africa [19]. Indeed, DENV-1 genotype V, DENV-2 cosmopolitan genotype and DENV-3 genotype III has over the years been the predominant circulating DENV in Africa [19].

Previous studies in Cameroon [48] and Ghana [17] revealed circulations of DENV-1 genotype V and DENV-2 genotype Cosmopolitan in febrile patients in Douala and Accra respectively.

Between 2016 and 2017 DENV-3/III re-emerged in Gabon, where the detected strain is closely related to those isolated in the same country in 2010. This highlight a stable circulation of this serotype in Gabon [49].

Interestingly studies highlights that DENV-3/III has the particularity to have a global distribution as consequence of its high transmissibility potential [50].

In contrast a different genotypic composition is observed by Ayolabi and colleagues during her study on DENV among febrile illnesses in Nigeria where DENV-1 (genotype I) and DENV-3 (genotype I) were identified as actively circulating in Lagos [19]. These results support a possible cryptic circulation of genotypes other than those predominantly detected in Africa.

Co-infection, with more than one serotype are likely to be much higher when multiple dengue serotypes co-circulate in a given population [51] and promoting the risk of emergence of recombinant virus strains that could have distinct properties [52].

Classically serotyping was performed in the lab by using gel based semi-nested conventional RT-PCR described by Lanciotti and colleagues [13]. The use of the CprM gene for dengue serotyping is known to be fast, economically cheaper [22] and sensitive [13], but these approaches require multiple handling steps (semi-nested PCR) which increase the risk of false-positive results due to amplicons contamination [12]. For the reasons highlighted below the Tib-Molbiol qRT-PCR system used during our study allow the simultaneous dengue virus serotyping in a single tube using 5 µl RNA input is a powerful and reliable tool for rapid dengue serotyping with lower risk of contamination.

Our study had several limitations. Firstly, we were limited by the number of isolates identified. This is probably due to the fact that the study is only focused on years 2017 and 2018. Secondly, collected sample were stored at + 4 °C and shipped on a weekly basis. This has the potential to impact considerably the viral isolation by impairing virus integrity. Thirdly, DENV-3 appears to be widespread but we must consider a potential sampling bias in favor of this serotype. Finally, processed isolates during this study were derived from sera samples collected in a single site at a given area, our result may not be representative of the real dengue burden and serotype distribution profile within the region.

Early management of dengue positive cases is key to reduce fatal outcomes, timely serotyping can provide early warning of dengue epidemics to improve management of patients and outbreaks [53]. Our aim is to adapt and evaluate the Tib-Molbiol, into flexible and portable qRT-PCR devices, as useful tool for DENV detection/serotyping near to the points of needs.

Our study highlights the importance of continuous molecular surveillance of arthropod-borne virus, particularly DENV, since the spread of emerging pathogens occurs rapidly between distant locations. This work will constitute a reference for future studies on DENV disease, dynamics of circulating strains and their impact on the virus epidemiology in Senegal.

## Conclusion

In summary, the present study describes the genetic diversity of dengue virus in Senegal between 2017 and 2018 at the serotype and genotype level using isolates available at the WHOCC and sequences from Senegal covering the study period available on Genbank. Overall, our results show a circulation of three dengue virus serotypes belonging to three genotypes during the study period and show a spatial distribution pattern of different serotypes marked by localization of serotype 2 isolates mainly in the north of the country, DENV-1 in Louga, Fatick and Thiès and a widespread of DENV-3 around the country.

However, the iceberg effect for DENV infection is well-known; cases reported to the surveillance system only represent a small proportion of total infections. To assess the true incidence and evaluate the level of herd immunity, a nationwide seroprevalence study is urgently needed.

## Supplementary Information

**Additional file 1.** Additional figures and tables.

## Data Availability

The datasets supporting the conclusions of this article are included within the article and its tables and figures. Additional data may be available from the corresponding author upon reasonable request.
