# Supplementary material for: Multifoci and multiserotypes circulation of dengue virus in Senegal between 2017 and 2018
Source: BMC Infect Dis. 2021 Aug 24;21:867. doi: 10.1186/s12879-021-06580-z (PMC8383925; doi:10.1186/s12879-021-06580-z)
Supplement: Supplementary file 1 — Additional file 1. Additional figures and tables. [file 12879_2021_6580_MOESM1_ESM.docx]

*BMC infectious disease*

Article

**Multifoci and Multiserotypes circulation of Dengue virus in Senegal between 2017 and 2018**

Idrissa Dieng^1*^, Marie Henriette Dior Ndione^1,^**^†^**, Cheikh Fall^1,^**^†^**, Moussa Moïse Diagne^1^, Mamadou Diop^2^, Aboubacry Gaye^2^, Mamadou Aliou Barry^2^, Boly Diop^3^, Mamadou Ndiaye^3^, Abdoulaye Bousso^3^, Gamou Fall^1^, Cheikh Loucoubar^2^, Oumar Faye^1^, Amadou Alpha Sall^1^ & Ousmane Faye^1^

1. Arboviruses and Haemorrhagic Fever Viruses Unit, Virology Department, Institut Pasteur de Dakar, Dakar 220, Senegal; Idrissa.DIENG@pasteur.sn (I.D.); Marie.NDIONE@pasteur.sn (M.H.D.N.);Cheikh.Fall@pasteur.sn(C.F.) MoussaMoise.DIAGNE@pasteur.sn (M.M.D.); Gamou.Fall@pasteur.sn(G.F.);Ousmane.FAYE@pasteur.sn (O.F.); Oumar.FAYE@pasteur.sn (O.F.); Amadou.SALL@pasteur.sn (A.A.S.)
2. Epidemiology, Clinical Research and Data Science Unit, Institut Pasteur de Dakar, Dakar 220, Senegal; Mamadou.Diop@pasteur.sn (M.D.); Aboubacry.Gaye@pasteur.sn (A.G.); Aliou.barry@pasteur.sn (M.A.B.);Cheikh.loucoubar@pasteur.sn (C.L.)
3. Prevention Department, Ministry of Health, Dakar, Senegal ; diopboly@yahoo.fr (B.D.); mamamorph@yahoo.fr (M.N.); abousso@cousenegal.sn (A.B.)

***** Correspondence: idrissa.DIENG@pasteur.sn; Tel.: +221-77-250-61-31

† These authors contributed equally to this work.

**Suppplementary files**

**Figure S1:** Barplot described the number and distribution of circulating serotype between 2017-2018 according to their sampling’s locations. Serotype 1 is colored in yellow, Serotype 2 in light blue and the serotype 3 in green.

**Figure S2:** Maximum likelihood (ML) phylogenetic tree based on CprM gene assessing genotypes of Senegalese DENV-1 strains. The tree was drawn with Iqtree (21) using the evolutionary model (TNe+G4). Each tips are colored according to the genotype. 1000 replicates of the sequences data were used for the robustess. The tree include 5 described DENV-1 genotypes. CprM sequences from Senegal are colored in dark green and belong to the genotype V (light green) Bootstrap confidence cut off are shown at each node

**Figure S3:** Maximum likelihood (ML) phylogenetic tree based on CprM gene assessing genotypes of Senegalese DENV-2 strains. The tree was drawn with Iqtree (21) using the evolutionary model (TIM3e+G4). Each tips are colored according to the genotype. 1000 replicates of the sequences data were used for the robustess. The tree include 6 described DENV-2 genotypes. CprM sequences from Senegal are colored in dark yellow and belong to the genotype IV (light yellow). Bootstrap confidence cut off are shown at each node.

**Figure S4:** Maximum likelihood (ML) phylogenetic tree based on CprM gene assessing genotypes of Senegalese DENV-3 strains. The tree was drawn with Iqtree (21) using the evolutionary model (K2P+G4). Each tips are colored according to the genotype. 1000 replicates of the sequences data were used for the robustess. The tree include 4 described DENV-3 genotypes. CprM sequences from Senegal are colored in dark purple and belong to the genotype IV (light purple). Bootstrap confidence cut off are shown at each node.

**Table S1:** Description of used Senegalese strains during this study. The “-” indicate that the corresponding informations for this strain were not available. DwoWS in clinical manifestations columns correspond to dengue infection without warming sign.

| **Sample name** | **Location** | **Sample type** | **Patient Age** | **Gender** | **Clinical Manifestations** | **Source** |
| --- | --- | --- | --- | --- | --- | --- |
| **297914** | Louga | C6-36 isolates | 31 | F | DwoWS | This Study |
| **297918** | Louga | C6-36 isolates | 27 | F | DwoWS | This Study |
| **297919** | Louga | C6-36 isolates | 15 | F | DwoWS | This Study |
| **297920** | Louga | C6-36 isolates | 32 | M | DwoWS | This Study |
| **297922** | Louga | C6-36 isolates | 24 | M | DwoWS | This Study |
| **298272** | Louga | C6-36 isolates | 12 | M | DwoWS | This Study |
| **298276** | Louga | C6-36 isolates | 28 | F | DwoWS | This Study |
| **298278** | Louga | C6-36 isolates | 13 | M | DwoWS | This Study |
| **298282** | Louga | C6-36 isolates | 25 | M | DwoWS | This Study |
| **298370** | Louga | C6-36 isolates | - | - | DwoWS | This Study |
| **298385** | Louga | C6-36 isolates | 27 | M | DwoWS | This Study |
| **314058** | Fatick | C6-36 isolates | 14 | F | DwoWS | This Study |
| **310045** | **Thies** | C6-36 isolates | **-** | - | DwoWS | This Study |
| **310395** | Richard Toll | C6-36 isolates | **20** | F | DwoWS | This Study |
| **310405** | Rosso | C6-36 isolates | **65** | F | DwoWS | This Study |
| **310411** | Rosso | C6-36 isolates | **-** | M | DwoWS | This Study |
| **310245** | Rosso | C6-36 isolates | **23** | F | DwoWS | This Study |
| **310402** | Rosso | C6-36 isolates | **38** | M | DwoWS | This Study |
| **297935** | Bokidiawe | C6-36 isolates | **47** | M | DwoWS | This Study |
| **309593** | Rosso | C6-36 isolates | **45** | M | DwoWS | This Study |
| **310324** | Matam | C6-36 isolates | **24** | M | DwoWS | This Study |
| **310398** | **Richard-Toll** | C6-36 isolates | **40** | F | DwoWS | This Study |
| **314109** | Fatick | C6-36 isolates | **29** | M | DwoWS | This Study |
| **310222** | Richard-Toll | C6-36 isolates | **25** | F | DwoWS | This Study |
| **316225** | **Touba** | C6-36 isolates | **11** | F | DwoWS | This Study |
| **316571** | **Touba** | C6-36 isolates | **31** | M | DwoWS | This Study |
| **316385** | **Touba** | C6-36 isolates | **-** | M | DwoWS | This Study |
| **316177** | **Touba** | C6-36 isolates | **20** | F | DwoWS | This Study |
| **316123** | **Touba** | C6-36 isolates | **18** | F | DwoWS | This Study |
| **316133** | **Touba** | C6-36 isolates | **22** | M | DwoWS | This Study |
| **316702** | **Touba** | C6-36 isolates | **13** | M | DwoWS | This Study |
| **310048** | Richard-Toll | C6-36 isolates | **20** | F | DwoWS | This Study |
| **310327** | Tambacounda | C6-36 isolates | **18** | F | DwoWS | This Study |
| **316206** | Touba | C6-36 isolates | **18** | M | DwoWS | This Study |
| **310279** | Dakar | C6-36 isolates | **-** | - | DwoWS | This Study |
| **316476** | Touba | C6-36 isolates | **30** | F | DwoWS | This Study |
| **316121** | Touba | C6-36 isolates | **25** | F | DwoWS | This Study |
| **310374** | Thies | C6-36 isolates | **-** | M | DwoWS | This Study |
| **310068** | Koki | C6-36 isolates | **30** | F | DwoWS | This Study |

**Table 1 (Continued):** Description of used Senegaleses strains during this study. The “-”indicates that the corresponding informations for this strain were not available

| **Sample name** | **Location** | **Sample type** | **Patient Age** | **Gender** | **Clinical Manifestations** | **Source** |
| --- | --- | --- | --- | --- | --- | --- |
| **MW288025.1** | Thies | Human Plasma | - | - | - | Genbank |
| **MW288026.1** | Thies | Human Plasma | - | - | - | Genbank |
| **MW288027.1** | Thies | Human Plasma | - | - | - | Genbank |
| **MW288028.1** | Thies | Human Plasma | - | - | - | Genbank |
| **MW288031.1** | Thies | Human Plasma | - | - | - | Genbank |
| **MW288033.1** | Thies | Human Plasma | - | - | - | Genbank |
| **MW288035.1** | Thies | Human Plasma | - | - | - | Genbank |
| **MW288037.1** | Thies | Human Plasma | - | - | - | Genbank |
| **MW288038.1** | Thies | Human Plasma | - | - | - | Genbank |
| **MW288039.1** | Thies | Human Plasma | - | - | - | Genbank |
| **MW288040.1** | Thies | Human Plasma | - | - | - | Genbank |
| **MW288032.1** | Thies | Human Plasma | - | - | - | Genbank |
| **MW288032.1** | Thies | Human Plasma | - | - | - | Genbank |
| **MW288024.1** | **Thies** | Human Plasma | **-** | - | - | Genbank |
| **MW288029.1** | **Thies** | Human Plasma | **-** | - | - | Genbank |
| **MW288030.1** | **Thies** | Human Plasma | **-** | - | - | Genbank |
| **MW288034.1** | **Thies** | Human Plasma | **-** | - | - | Genbank |

**Table S2:** Summary table of used Senegalese DENV isolates during this study with their sampling locations, years of sampling and Serotypes assignement using CprM sequencing followed by Phylogenetic Analysis (Column 4) and/or Tib-Molbiol qRT-PCR serotyping tool. NA (Not Available) means that qRT-PCR serotyping was not performed for this particular strains since that sequences were downloaded directly from Genbank

| Strains | Sampling locations | Years | Serotypes Assignment | |
| --- | --- | --- | --- | --- |
|  |  |  | Phylogenetic | Tib-Molbiol |
| **297914** | Louga | 2017 | Serotype 1 | Serotype 1 |
| **297918** | Louga | 2017 | Serotype 1 | Serotype 1 |
| **297919** | Louga | 2017 | Serotype 1 | Serotype 1 |
| **297920** | Louga | 2017 | Serotype 1 | Serotype 1 |
| **297922** | Louga | 2017 | Serotype 1 | Serotype 1 |
| **298272** | Louga | 2017 | Serotype 1 | Serotype 1 |
| **298276** | Louga | 2017 | Serotype 1 | Serotype 1 |
| **298278** | Louga | 2017 | Serotype 1 | Serotype 1 |
| **298282** | Louga | 2017 | Serotype 1 | Serotype 1 |
| **298370** | Louga | 2017 | Serotype 1 | Serotype 1 |
| **298385** | Louga | 2017 | Serotype 1 | Serotype 1 |
| **314058** | Fatick | 2018 | Serotype 1 | Serotype 1 |
| **310045** | Rosso | 2018 | Serotype 2 | Serotype 2 |
| **310395** | Richard Toll | 2018 | Serotype 2 | Serotype 2 |
| **310405** | Rosso | 2018 | Serotype 2 | Serotype 2 |
| **310411** | Rosso | 2018 | Serotype 2 | Serotype 2 |
| **310245** | Rosso | 2018 | Serotype 2 | Serotype 2 |
| **310402** | Rosso | 2018 | Serotype 2 | Serotype 2 |
| **297935** | Bokidiawe | 2017 | Serotype 2 | Serotype 2 |
| **309593** | Rosso | 2018 | Serotype 2 | Serotype 2 |
| **310324** | Matam | 2018 | Serotype 2 | Serotype 2 |
| **310398** | Rosso | 2018 | Serotype 2 | Serotype 2 |
| **314109** | Fatick | 2018 | Serotype 3 | Serotype 3 |
| **310222** | Richard Toll | 2018 | Serotype 3 | Serotype 3 |
| **316225** | Touba | 2018 | Serotype 3 | Serotype 3 |
| **316571** | Touba | 2018 | Serotype 3 | Serotype 3 |
| **316385** | Touba | 2018 | Serotype 3 | Serotype 3 |
| **316177** | Touba | 2018 | Serotype 3 | Serotype 3 |
| **316123** | Touba | 2018 | Serotype 3 | Serotype 3 |

**Table S2 (Continued):** Summary table of used Senegalese DENV isolates during this study with their sampling locations, years of sampling and Serotypes assignement using CprM sequencing followed by Phylogenetic Analysis (Column 4) and/or Tib-Molbiol qRT-PCR serotyping tool. NA (Not Available) means that qRT-PCR serotyping was not performed for this particular strains since that sequences were downloaded directly from Genbank

| Strains | Sampling locations | Years | Serotypes Assignment | |
| --- | --- | --- | --- | --- |
|  |  |  | Phylogenetic | Tib-Molbiol |
| **316133** | Touba | 2018 | Serotype 3 | Serotype 3 |
| **316702** | Touba | 2018 | Serotype 3 | Serotype 3 |
| **310048** | Richard Toll | 2018 | Serotype 3 | Serotype 3 |
| **310327** | Tambacounda | 2018 | Serotype 3 | Serotype 3 |
| **316206** | Touba | 2018 | Serotype 3 | Serotype 3 |
| **310279** | Dakar | 2018 | Serotype 3 | Serotype 3 |
| **316476** | Touba | 2018 | Serotype 3 | Serotype 3 |
| **316121** | Touba | 2018 | Serotype 3 | Serotype 3 |
| **310374** | Thies | 2018 | Serotype 3 | Serotype 3 |
| **310068** | Koki | 2018 | Serotype 3 | Serotype 3 |
| **MW288032.1** | Thies | 2018 | Serotype 1 | NA |
| **MW288036.1** | Thies | 2018 | Serotype 1 | NA |
| **MW288024.1** | Thies | 2018 | Serotype 2 | NA |
| **MW288030.1** | Thies | 2018 | Serotype 2 | NA |
| **MW288034.1** | Thies | 2018 | Serotype 2 | NA |
| **MW288029.1** | Thies | 2018 | Serotype 2 | NA |
| **MW288027.1** | Thies | 2018 | Serotype 3 | NA |
| **MW288025.1** | Thies | 2018 | Serotype 3 | NA |
| **MW288039.1** | Thies | 2018 | Serotype 3 | NA |
| **MW288031.1** | Thies | 2018 | Serotype 3 | NA |
| **MW288035.1** | Thies | 2018 | Serotype 3 | NA |
| **MW288038.1** | Thies | 2018 | Serotype 3 | NA |
| **MW288040.1** | Thies | 2018 | Serotype 3 | NA |
| **MW288033.1** | Thies | 2018 | Serotype 3 | NA |
| **MW288037.1** | Thies | 2018 | Serotype 3 | NA |
| **MW288028.1** | Thies | 2018 | Serotype 3 | NA |
| **MW288026.1** | Thies | 2018 | Serotype 3 | NA |

**Table S3 :** Dengue virus CprM gene sequences used in the serotype and genotype phylogenetic analysis

| Genbank Accession  Number | Year of isolation | Local of isolation | Serotype | Genotype |
| --- | --- | --- | --- | --- |
| Serotype phylogenetic analysis | | | | |
| LC379220.1 | 2014 | Ghana | DENV-3 | Genotype III |
| JN662391.1 | 2009 | China | DENV-3 | Genotype III |
| KF954947.1 | 2013 | China | DENV-3 | Genotype III |
| KM403591.1 | 2013 | Singapore | DENV-1 | Genotype V |
| KY057372.1 | 2012 | Indonesia | DENV-1 | Genotype I |
| KY627763.1 | 2016 | Burkina Faso | DENV-2 | Cosmopolitan  genotype |
| KX452042.1 | 2014 | Malaysia | DENV-2 | Cosmopolitan  genotype |
| KY924607.1 | 2016 | Viet Nam | DENV-4 | Genotype I |
| Genotype phylogenetic analysis | | | | |
| FJ410249 | 2008 | Viet Nam | DENV-1 | Genotype I |
| GQ868618 | 2003 | Cambodia | DENV-1 | Genotype I |
| AF309641 | 2002 | Myanmar | DENV-1 | Genotype I |
| KP406802 | 2005 | South Korea | DENV-1 | Genotype I |
| KJ755855 | 2013 | India | DENV-1 | Genotype I |
| KJ649286 | 2011 | Saudi Arabia | DENV-1 | Genotype I |
| AY726552 | 2002 | Myanmar | DENV-1 | Genotype I |
| AF180817 | 1964 | Thailand | DENV-1 | Genotype II |
| EF457905 | 1972 | Malaysia | DENV-1 | Genotype III |
| EF025110 | 2002 | China | DENV-1 | Genotype IV |
| FJ196842 | 2003 | China | DENV-1 | Genotype IV |
| KT827364 | 2001 | USA | DENV-1 | Genotype IV |
| AB189121 | 1998 | Indonesia | DENV-1 | Genotype IV |
| AB204803 | 2004 | Japan | DENV-1 | Genotype IV |
| DQ285560 | 2004 | Reunio | DENV-1 | Genotype IV |
| AB195673 | 2003 | Japan | DENV-1 | Genotype IV |
| JQ915077 | 2002 | New Caledonia | DENV-1 | Genotype IV |
| JQ915080 | 2010 | New Caledonia | DENV-1 | Genotype IV |
| KR919820 | 2015 | Brunei | DENV-1 | Genotype IV |
| MG053118 | 2016 | India | DENV-1 | Genotype V |
| JF815177 | 2010 | India | DENV-1 | Genotype V |
| KU509255 | 2011 | India | DENV-1 | Genotype V |
| KX380802 | 2012 | Singapore | DENV-1 | Genotype V |
| KM403584 | 2013 | Singapore | DENV-1 | Genotype V |
| KR024705 | 2014 | China | DENV-1 | Genotype V |
| AY732476 | 1980 | Thailand | DENV-1 | Genotype V |
| FJ850070 | 2000 | Brazil | DENV-1 | Genotype V |
| AY593213 | 1963 | India | DENV-1 | Genotype V |
| EU482591 | 2008 | USA | DENV-1 | Genotype V |
| AF038403.1 | 1944 | New Guinea | DENV-2 | Genotype I |
| AF204177.1 | 1989 | China | DENV-2 | Genotype I |
| AF204178.1 | 1987 | China | DENV-2 | Genotype I |
| AF022437.1 | 1999 | Japan | DENV-2 | Genotype II |
| AF100464.1 | 1996 | Thailand | DENV-2 | Genotype II |
| AF469176.1 | 1998 | China | DENV-2 | Genotype II |
| M20558.1 | 1988 | Jamaica | DENV-2 | Genotype III |
| AF489932.1 | 1998 | Brazil | DENV-2 | Genotype III |
| GQ398314.1 | 1994 | Puerto Rico | DENV-2 | Genotype III |
| KY627763.1 | 2016 | Burkina Faso | DENV-2 | Cosmopolitan  genotype |
| AY858035.2 | 2004 | Indonesia | DENV-2 | Cosmopolitan  genotype |
| EU081180.1 | 2005 | Singapore | DENV-2 | Cosmopolitan  genotype |
| JX470186.1 | 2010 | China | DENV-2 | Cosmopolitan  genotype |
| KX577715.1 | 2015 | China | DENV-2 | Cosmopolitan  genotype |
| KY550240.1 | 2016 | India | DENV-2 | Cosmopolitan  genotype |
| AF100465.1 | 1987 | Venezuela | DENV-2 | Genotype V |
| AF100467.1 | 1995 | Peru | DENV-2 | Genotype V |
| AY593227.1 | 1974 | India | DENV-2 | Genotype V |
| AY593228.1 | 1956 | India | DENV-2 | Genotype V |
| AY744677 | 1989 | French Polynesia | DENV-3 | Genotype I |
| JQ920481 | 1989 | New Caledonia | DENV-3 | Genotype I |
| AY744683 | 1992 | French Polynesia | DENV-3 | Genotype I |
| FJ898456 | 1995 | Samao | DENV-3 | Genotype I |
| AY744685 | 1994 | French  Polynesia | DENV-3 | Genotype I |
| JQ920486 | 1996 | New Caledonia | DENV-3 | Genotype I |
| KC762685 | 2008 | Indonesia | DENV-3 | Genotype I |
| AY496879 | 1997 | Philippines | DENV-3 | Genotype I |
| DQ675519 | 1995 | Taiwan | DENV-3 | Genotype I |
| KU509279 | 2008 | Philippines | DENV-3 | Genotype I |
| AY648961 | 1978 | Indonesia | DENV-3 | Genotype I |
| AB189128 | 1998 | Indonesia | DENV-3 | Genotype I |
| KF955462 | 2001 | Cambodia | DENV-3 | Genotype II |
| FJ639719 | 2000 | Cambodia | DENV-3 | Genotype II |
| DQ675530 | 1998 | Taiwan | DENV-3 | Genotype II |
| KC261634 | 2012 | China | DENV-3 | Genotype II |
| KU509280 | 2011 | Thailand | DENV-3 | Genotype II |
| EU482458 | 2006 | Viet Nam | DENV-3 | Genotype II |
| LC379220.1 | 2014 | Ghana | DENV-3 | Genotype III |
| JN662391.1 | 2009 | China | DENV-3 | Genotype III |
| MG053149 | 2016 | India | DENV-3 | Genotype III |
| JN940919 | 2010 | India | DENV-3 | Genotype III |
| KF973481 | 2011 | Nicaragua | DENV-3 | Genotype III |
| JX669494 | 2005 | Brazil | DENV-3 | Genotype III |
| AY099337 | 1999 | Martinique | DENV-3 | Genotype III |
| JQ922555 | 1966 | India | DENV-3 | Genotype III |
| M93130 | 1956 | Philippines | DENV-3 | Genotype IV |
| AF317645 | 1980 | China | DENV-3 | Genotype IV |
